# 

**Published:** 1888-06-16

**Authors:** 


### CONTENTS.

**The Growth of Hospital Opinion** - 173

**A Plea for the London Hospital** - 174

**The Doctor's Pulpit.**—Tonics - 175

**Patients' Contributions to Hospitals.** By Mr. W. Burdett-Coutts, M.P. - 176

**The Mildmay Cottage Hospital** - 178

**Round About the Asylums.** VII. By a Medical Superintendent - 179

**Notes and News** - 180

**Everybody's Page** - 182

**Annotations.**—Sympathy and Congratulation.—The Care of Consumption.—The Case for Hanging - 183

**Medicine Men of the World** - 184

**An Ishmalite.** By Leslie Keith, Author of "The Chilcotes," "East and West," etc. - 185

**Scotch Physicians and Surgeons.**—James Syme - 186

**Amusements with Profit for all Ages.**—For Men and Women.—Children's Corner.—Puzzles, etc. - 188

**The Student's Column.**—"The Hall" Examination Papers - 188

**Hospitals and the Drama** - 188

**EXTRA SUPPLEMENT.—THE NURSING MIRROR.**

En Passant.—The Swansea Memorial.—Staffordshire Institution for Nurses.—The Provinces and the Pension Fund.—A Womanly Queen.—Cheap Nursing Lectures.—District Nursing in Belfast.—Thanks to the Matron.—The Glasgow Training Home - xli
Notes from America - xlii
Original Articles.—Old and New Nurses - xlii
Useful Hints - xliii
A District Matron's Diary.—A Week's Work - xliii
Everybody's Opinion.—The Patient Speaks.—Presence of Mind *v.* Panic - xliv
Appointments.—Vacancies.—Exchange and Mart - xliv

